# Esophageal cancer patients’ need for information and support in making a treatment decision between standard surgery and active surveillance

**DOI:** 10.1002/cam4.6308

**Published:** 2023-07-01

**Authors:** Merel Hermus, Berend J. van der Wilk, Rebecca Chang, Jan Willem T. Dekker, Peter‐Paul L. O. Coene, Grard A. P. Nieuwenhuijzen, Camiel Rosman, Joos Heisterkamp, Henk H. Hartgrink, Liesbeth Timmermans, Bas P. L. Wijnhoven, Charlène J. van der Zijden, Jan J. B. van Lanschot, Jan Busschbach, Sjoerd M. Lagarde, Leonieke W. Kranenburg

**Affiliations:** ^1^ Department of Psychiatry, Section of Medical Psychology and Psychotherapy Erasmus University Medical Center Rotterdam The Netherlands; ^2^ Department of Surgery Erasmus Cancer Institute, Erasmus University Medical Center Rotterdam The Netherlands; ^3^ Department of Surgery Reinier de Graaf Hospital Delft The Netherlands; ^4^ Department of Surgery Maasstad Hospital Rotterdam The Netherlands; ^5^ Department of Surgery Catharina Hospital Eindhoven The Netherlands; ^6^ Department of Surgery Radboud University Medical Center Nijmegen The Netherlands; ^7^ Department of Surgery Elisabeth Tweesteden Hospital Tilburg The Netherlands; ^8^ Department of Surgery Leiden University Medical Center Leiden The Netherlands; ^9^ Department of Primary and Community Care Radboud University Medical Center Nijmegen The Netherlands

**Keywords:** active surveillance, esophageal cancer, esophagectomy, experimental treatment, information needs, shared decision‐making, supportive needs

## Abstract

**Background:**

This study explores patients’ need for information and support in deciding on esophageal cancer treatment, when experimental active surveillance and standard surgery are both feasible.

**Methods:**

This psychological companion study was conducted alongside the Dutch SANO‐trial (Surgery As Needed for Oesophageal cancer). In‐depth interviews and questionnaires were used to collect data from patients who declined participation in the trial because they had a strong preference for either active surveillance (*n* = 20) or standard surgery (*n* = 20). Data were analyzed using both qualitative and quantitative techniques.

**Results:**

Patients prefer to receive information directly from their doctors and predominantly rely on this information to make a treatment decision. Other information resources are largely used to confirm their treatment decision. Patients highly value support from their loved ones and appreciate emphatic doctors to actively involve them in the decision‐making process. Overall, patients’ needs for information and support during decision‐making were met.

**Conclusions:**

The importance of shared decision‐making and the role doctors have in this process is underlined. The role of doctors is essential at the initial phase of decision‐making: Once patients seem to have formed their treatment preference for either active surveillance or surgery, the influence of external resources (including doctors) may be limited.

## 1 INTRODUCTION

Shared decision‐making is seen as the pinnacle of patient‐centered care, in which clinicians and patients work together toward a treatment decision that best suits the patients’ individual needs and lifestyle.^1^, ^2^ For patients to actively contribute and be satisfied with their treatment choice, they need to have sufficient information about the pros and cons of available treatment options.

It can be challenging to make a well‐informed decision on treatment when considerations of a range of diagnostic, therapeutic, and prognostic uncertainties and associate preferences are involved.^3^ This is currently the case for esophageal cancer. Standard curative care for esophageal cancer consists of neoadjuvant chemoradiotherapy (nCRT) followed by surgery. Currently, the Dutch SANO‐trial (Surgery As Needed for Oesophageal cancer) examines whether nCRT followed by active surveillance is non‐inferior to nCRT followed by standard surgery for patients in whom no vital tumor cells can be detected with diagnostics in clinical response evaluations, 3 months after nCRT (i.e., clinically complete responders).^4^ During active surveillance, patients undergo frequent systematic clinical response evaluations after nCRT and surgical resection is only performed when locoregional regrowth of cancer—in the absence of distant dissemination—is proven or highly suspected. Although a recent meta‐analysis showed that patients on active surveillance have overall survival rates comparable to patients who underwent standard surgery, the number of patients who underwent active surveillance treatment is limited and definite prospective data are still awaited to demonstrate the non‐inferiority of this treatment.^5^, ^6^, ^7^ This results in a novel treatment decision for patients between “standard treatment” or “experimental treatment”: A typical situation for shared decision‐making wherein patients have to weigh possible outcomes, taking into account a certain level of uncertainty.

The present study is the first to focus on esophageal cancer patients’ need for information and support during decision‐making when active surveillance is introduced as a (still experimental) treatment alternative.^8^, ^9^

## 2 METHODS

This present study is a psychological companion study to the Dutch SANO‐trial. In this stepped‐wedge, cluster randomized controlled trial, patients with a clinically complete response undergo either active surveillance or standard esophagectomy, depending on which study arm the participating hospital was recruiting for.^4^ Some patients declined participation in the SANO‐trial because they preferred the opposed treatment than was offered. For example, participation could mean that patients were assigned to active surveillance whereas they had a preference for standard surgery (or vice versa). These patients therefore refused participation in the SANO‐trial and were consecutively invited to participate in the present “NO‐SANO” study.

Patients were offered extensive written study information prior to nCRT. One week after being invited for study participation by their surgeon, patients were phoned to provide additional explanation about the study if needed, and to ask for their decision to participate in the study. Recruitment took place in seven participating hospitals in the Netherlands, and data collection occurred from January 2019 to May 2020. Recruitment continued until data saturation for the qualitative study part was reached.^10^, ^11^ All patients gave written informed consent.

We report on those patients’ need for information and support during the decision‐making process. For this purpose, both qualitative and quantitative methods were used. Data were collected either 3 months after completion of nCRT or, if applicable, before surgery was performed.

In‐depth interviews were conducted by using a predefined list. Interviews were conducted in the hospital or at the patients’ home, depending on the patients’ preference. All interviews were audio‐recorded and transcribed verbatim. Data were analyzed according to the principles of grounded theory. This method was chosen because it suits the explorative nature and aim of this study.^12^ To ensure the reliability and robustness of data‐analysis triangulation was applied. Data were imported and analyzed using NVivo software, version 20.3.1.

Questionnaires were administered. Health literacy was assessed with screening items that are effective in detecting patients with limited health literacy.^13^, ^14^, ^15^ Information needs were assessed with two statements: “I want to receive as much information as possible about my disease and treatment” and “I prefer to make important decisions about the treatment completely by myself.” Patients ranked these statements on a 100 mm VAS‐scale ranging from “completely disagree” to “completely agree.” Additionally, patients were asked how they prefer to receive information about treatment options, with multiple answers allowed. For data analysis, we used independent samples *t*‐test (with α set at 0.05) to make comparisons between groups. Data were analyzed using IBM SPSS Statistics, version 25. The Erasmus MC Medical Ethical Committee approved the study (MEC‐2018‐1526).

## 3 RESULTS

Forty patients were enrolled in the study. Twenty patients preferred active surveillance (40% female, median age = 70.5 years, *SD* = 7.0) and 20 patients preferred standard surgery (25% female, median age = 62, *SD* = 8.7).

### 3.1 Interviews

We identified three central concepts: (1) The overall experience of the decision‐making process, (2) information resources, and (3) patients’ ideas on how to improve guidance in the decision‐making process for future patients.

#### 3.1.1 The overall decision‐making process

Most patients quickly made up their mind to opt for either active surveillance or standard surgery. Patients who experienced doubts in the decision‐making process, mentioned that they always knew deep down inside what treatment to opt for. Once the decision was final, all patients displayed confidence in their choice.

The statement “my body my choice” was often given to emphasize that they were the ones to make the final decision. However, patients highly valued the full support they received from their partners about their treatment choice. This support was valuable because it felt like a validation of their treatment decision.

Patients opting for active surveillance seem to rely on a “gut feeling” when making a treatment decision. They feel confident in opting for active surveillance, because this treatment “feels” as the best option.

It is purely a matter of feeling, because if doctors say ‘everything looks really good’ then I cannot imagine that there are people who decide: ‘please take this thing [the esophagus] out!

Patients opting for surgery seem to rely on the established treatment instead of the experimental treatment; they have confidence in the surgical expertise and experience of their doctor.

they know what they do because they routinely perform this surgery.

I trust more in the doctors’ expertise than just taking information from someone who might just be very afraid of the consequences of surgery.

#### 3.1.2 Information resources

The main used source for health information was the information patients received at the hospital.

I trust the doctors, I read all information they give to me, and I do not need to read about others’ experiences

Patients repeatedly mentioned a book recommended by their doctor, about a true story of a journalist who underwent esophagectomy. Despite the recommendation, most patients did not want to read this book.

You just don't know how it will turn out for you; it is such a personal experience.

Patients found support in talking to others in the decision‐making process. As stated earlier, most conversations were aimed at receiving support, instead of actually asking for advice.

Regarding the use of internet, opinions were divided among patients. In the active surveillance group, some consulted websites of patient associations to gain more insight in quality of life after surgery. This confirmed their decision for active surveillance, because the images and stories they found there, scared them off surgery. On the other hand, others in the active surveillance group choose to avoid the internet to protect themselves from an overload of information and the uncertainty about which information is reliable.

Also in the surgery group, some patients consulted the internet. For them, the main goal was to obtain statistical information. They did avoid reading about other patients’ personal experiences with surgery.

I want to minimize risk. So, I want to know what the risks are of postponing surgery and if I will still be on time if I decide to undergo surgery later on.

I absolutely do not use Google, you will find stories that are even worse than the reality

#### 3.1.3 Patients’ ideas on how to improve guidance in the decision‐making process

Patients were generally satisfied with the provided care. Overall, patients attach great value to emotional support: personal attention, consideration of personal feelings, and honest communication The following skills were appreciated in doctors: a (non‐verbal) calm and relaxed attitude; showing respect for the treatment decision made; being a good listener; answering questions; thinking along; being straight to the point; being objective by not steering into a direction in the decision‐making; and using comprehensible language.

“It would be nice if there was more attention being paid to the feelings of patients”.

Patients stressed the importance of doctors equally explaining both treatment options as good as possible and subsequently leaving the final decision up to the patient. So, they want their doctor to guide them in the decision‐making, but not to make the final decision

Considering guidance, a significant group emphasized they would appreciate decision counseling.

I think it would be nice if there was a separate appointment to pay attention to what is important to the patient, to give a better guidance in making a decision.

### 3.2 Questionnaires

Table 1 shows the level of health literacy measured. Table 2 shows preferences about information provision. There were no statistically significant differences between the groups. The VAS‐scales on information needs resulted in high scores on the statement “I want to receive as much information about the disease and treatment as possible” (M = 82.89, SD = 19.44) and great variability on the statement “I prefer to make treatment decisions by myself”, as reflected by the high standard deviation (M = 49.05, SD = 23.12).

**TABLE 1:** Level of health literacy measured by two statements (*N*=39).^13^, ^14^, ^15^

	Frequency	Valid %	Cumulative %
How often do you have someone help you read hospital materials?			
Always	5	13.5	13.5
Often	2	5.4	18.9
Sometimes	6	16.2	35.1
Occasionally	9	24.3	59.5
Never	15	40.5	100.0
Total	37	100.0	
Missing	4		
How often do you have problems learning about your medical condition because of difficulty reading hospital materials?			
Always	‐	‐	‐
Often	3	8.1	8.1
Sometimes	3	8.1	16.2
Occasionally	5	13.5	29.7
Never	26	70.3	100.0
Total	37	100.0	
Missing	4		

**TABLE 2:** Patient responses to how they prefer to receive information about treatment options (*N*=39).

	%
How do you prefer to receive information?	
From the doctor	82.9
From the nurse	51.2
From any specialist, doesn't matter which one	48.8
Written information	29.3
From the internet, written	9.8
From the internet, video	2.4
From patient organizations	4.9

## 4 DISCUSSION AND CONCLUSION

This study shows that once patients have decided what treatment to opt for, patients are confident and are favoring information that is confirming their decision.

In general, patients indicate that they want to receive as much information about their disease and treatment options as possible, and preferably from their doctor. The reported levels of health literacy underline the importance of ensuring that patients also comprehend all this information in order to make appropriate health decisions.

Patients who opt for active surveillance seem to base their decision on a “feeling” that it is the right thing to do. Patients who opt for surgery, seem to base their decision rather on a reliance on their doctors’ surgical expertise and finding reassurance in choosing the established treatment. Regardless of treatment preference, resources outside the hospital, such as the Internet and conversations with others, are not the preferred source of information. This particular preference may be influenced by the relatively older age of the participants, as older individuals may face challenges in accessing information online.^16^ Additionally, older generations tend to put more value on the doctor's insights and advice.^17^ However, the mean age of our sample is representative of the average esophageal cancer patient.

Patients tend not to actively search for information to help them *make* a treatment decision, rather they search for information that *confirms* the treatment decision they already made. This observation is known as “confirmation bias”: The tendency to prefer information that confirms one's initial beliefs.^18^, ^19^ The search for confirmation can be extended to supportive needs. Patients use the support they receive from their loved ones as a confirmation they made the right decision.

All patients are aware that they are faced with a personal decision: They substantiate their decision with “my body my choice,” and they emphasize that doctors should leave the final decision up to the patient. However, patients do want a doctor to guide them through the decision‐making process by providing well‐balanced information, while providing emotional support. It is only *after* this guiding process patients seem to differ in how they prefer the decision is made: alone, together, or to leave it completely up to the doctor.

The treatment decision requires a subjective cost–benefit evaluation wherein the patients’ individual preconceptions about a treatment choice may influence the information filtering process.^20^ Such preconceptions can make it harder for (treatment) information carrying opposite values, to get through in the filtering process.^21^ Individuals tend to reject information that is contradictory to their beliefs and values, in order to protect their sense of worth that people derive from their existing values. In addition, the filtering of information is also subject to the extent in which individuals evaluate the source of information as trustworthy. Our study results show that patients indeed experienced the doctors’ information as a trusted source (e.g., compared to the Internet). Information from the doctor may go beyond factual information, and patients may pay close attention to more subtle or non‐verbal cues. In another study by our group, we found that this indeed was the case.^22^ Next to information from medical professionals, patients may also consider stories from their family or friends as a trusted source of information, and take this into account, even if it may not correspond with information provided by their doctor. Last but not least, the filtering of information is further influenced by the patients’ emotions. That is, patients do not necessarily make “rational” choices. This was clearly demonstrated by another study from our group, where we found that several patients with a complete clinical response after nCRT worded their motivation for active surveillance treatment as “I feel all good right now, I trust my body, why would I undergo surgery“; and some patients opting for surgery motivated their choice with “I know it may not be necessary to undergo surgery, but I would not forgive myself if I did not do everything that may possibly help”, indicating anticipated regrets.^23^ So, in short, the final outcome (i.e., treatment choice) then is the result of a subjective evaluation of multiple factors. As this can be quite complex, presuming that “just providing the facts” will lead to an well‐considered treatment choice is obsolete.^24^ Instead, it is preferable to explicitly include patients’ preferences and values in the choice for a certain type of treatment, as is appropriate to the theory of shared decision‐making.^25^ Working from such a practical model, with an eye on patient's individual values, also helps to address the confirmation bias. Discussing what matters to patients and why, may open up the way to alternative points of view, without patients feeling overridden or compromised in their sense of self‐worth.

A limitation of this study is that only patients with strong treatment preferences were included. However, if even patients with strong preferences want to be guided in the decision‐making process, this will not be different for patients without a strong preference.

This study underlines the important guiding role of health professionals in the decision‐making process for esophageal cancer patients to decide between experimental active surveillance and standard surgery. Health professionals should be aware that their role is most crucial at the initial phase of the decision‐making process. At this phase, patients’ treatment preference is formed and thus should information provision at this stage be as complete and objective as possible. All information provision later on in the process seem to be less influential for the final decision and more filtered to confirm the treatment preference already formed.

## AUTHOR CONTRIBUTIONS

**Merel Hermus:** Data curation (equal); formal analysis (equal); writing – original draft (equal). **Berend J. van der Wilk:** Conceptualization (equal); data curation (equal); writing – review and editing (equal). **Rebecca Chang:** Data curation (equal); formal analysis (equal). **Jan Willem T. Dekker:** Resources (equal); writing – review and editing (equal). **Peter‐Paul L.O. Coene:** Resources (equal); writing – review and editing (equal). **Grard A.P. Nieuwenhuijzen:** Resources (equal); writing – review and editing (equal). **Camiel Rosman:** Resources (equal); writing – review and editing (equal). **Joos Heisterkamp:** Resources (equal); writing – review and editing (equal). **Henk H. Hartgrink:** Resources (equal); writing – review and editing (equal). **Liesbeth Timmermans:** Conceptualization (equal); funding acquisition (equal). **Bas P.L. Wijnhoven:** Resources (equal); writing – review and editing (equal). **Charlène J. van der Zijden:** Writing – review and editing (equal). **Jan J.B. van Lanschot:** Conceptualization (equal); writing – review and editing (equal). **Jan Busschbach:** Conceptualization (equal); writing – review and editing (equal). **Sjoerd M. Lagarde:** Conceptualization (equal); funding acquisition (equal); project administration (equal); resources (equal); writing – review and editing (equal). **Leonieke W. Kranenburg:** Conceptualization (equal); data curation (equal); formal analysis (equal); funding acquisition (equal); supervision (equal); writing – review and editing (equal).

## FUNDING INFORMATION

Maag Lever Darm Stichting ZP‐18‐13.

## CONFLICT OF INTEREST STATEMENT

The authors declare no conflict of intrest.

## ETHICS STATEMENT

The Erasmus MC Medical Ethical Committee approved the study (MEC‐2018‐1526).

## PATIENT CONSENT STATEMENT

All participants provided written informed consent.

## Data Availability

The data that support the findings of this study are available from the corresponding author upon reasonable request.
